# Papaya (*Carica papaya* L.) seed as a potent functional feedstuff for poultry – A review

**DOI:** 10.14202/vetworld.2020.1613-1619

**Published:** 2020-08-17

**Authors:** Sugiharto Sugiharto

**Affiliations:** Department of Animal Science, Faculty of Animal and Agricultural Sciences, Diponegoro University, Tembalang Campus, Semarang, Central Java, Indonesia

**Keywords:** antimicrobial, antioxidant, antiparasitic, growth promoter, papaya by-product, protein-rich feedstuff

## Abstract

The steady increase in the price of protein feed ingredients and the retraction of antibiotics from diets has encouraged nutritionists to search the alternatives for protein source and functional feedstuffs that can substitute the role of antibiotic growth promoters in poultry production. With crude protein of 24-30%, in vitro protein digestibility of 80% and proportion of essential amino acids of 47%, seed from ripe papaya may be exploited as the alternative protein feed ingredient for poultry. Moreover, the growth promoting effect, antimicrobial and antiparasitic activities, and immunomodulatory and antioxidative activities may confirm the potential of papaya seed as a functional feedstuff that could replace the role of antibiotic growth promoters for poultry. The in-depth study is needed to further elucidate the functionalities of papaya seed onpoultry. This review provides the updates on the nutritional contents of papaya seed, the potential of papaya seed as an alternative to conventional protein-rich ingredient, the growth-promoting effect of feeding papaya seed, the antimicrobial and antiparasitic activities of papaya seed, antioxidative activities of papaya seed, and the immunomodulatory activity of papaya seed on poultry.

## Introduction

Agro-industrial by-products have recently attracted a growing interest from the nutritionists as feed ingredients for poultry. Dealing with environmental pollution and high cost of poultry feed, the utilization of agro-industrial by-products as feed ingredients is beneficial for the environmental health and sustainable poultry production [1]. Papaya (*Carica papaya* L.) seed is one of the agro-industrial by-products that have been included in poultry rations, due to its substantial amounts of crude protein, fat, and ash [2]. Papaya seed also contains some functional properties that may act as growth promoters [3], antimicrobial and antiparasitic factors [4-6], and immunomodulatory and anti-inflammatory agents [7,8] for poultry. Taking these facts into consideration, papaya seed may be used as feed ingredient to reduce feed cost as well as functional feedstuff to improve the health and well-being of poultry.

Papaya is a tropical fruit that is available throughout the year [9]. Papaya fruit is commonly consumed fresh as a dessert or juice. Papaya seeds are black in color and embedded in the fruit pulp [7]. In general, the seed from ripe papaya represents of about 16% of the fresh fruit weight and is considered as a by-product. The abundant availability throughout the year and less economic value of papaya seed have encouraged the nutritionists to exploit such by-product as a protein-rich feed ingredient as well as functional feedstuff for poultry.

The updates regarding the nutritional contents of papaya seed, the potential of papaya seed as an alternative to conventional protein-rich ingredient, the growth promoting effect of feeding papaya seed, the antimicrobial and antiparasitic activities of papaya seed, antioxidative activities of papaya seed, and the immunomodulatory activity of papaya seed on poultry are presented in the present review.

## General Nutritional Compositions of Papaya Seed

The use of agro-industrial by-products in poultry rations has been encouraged due to the steady increase in price of conventional feedstuffs. Among the agro-industrial by-products, papaya seed has been explored extensively as the potential of feed ingredients for poultry. The latter by-product contains substantial amounts of crude protein, fat, and ash that may be utilized by the chickens. Table-1 [2,3,10-16] shows proximate contents of dried papaya seed meal. There are great variations in the proximate compositions of papaya seed. It seems that the differences in the varieties or cultivars, ripening stages, and climates may be responsible for the divergent proximate compositions of the dried papaya seed [10,17].

**Table-1:** Proximate contents of dried papaya seed meal.

References	Nutrients (%)			

	Crude protein	Crude fat	Crude fiber	Crude ash
Adesuyi and Ipinmoroti [2]	29.1-31.9	29.4-31.6	7.80-9.40	9.94-11.5
Muazu and Aliyu-Paiko [3]	26.3	28.2	30.8	2.09
dos Santos *et al*. [10]	28.6	29.7	8.78	6.94
Bolu *et al*. [11]	30.1	34.8	1.67	7.11
El-Safy *et al.* [12]	31.3	32.5	5.19	8.89
Maisarah *et al.* [13]	25.1	0.00	45.6	8.20
Azevedo and Campagnol [14]	25.4	21.0	24.3	6.43
Moses and Olanrewaju [15]	27.4	28.6	8.02	5.21
Seshamamba *et al.* [16]	33.0	NM	18.8	9.00

NM: Not measured

Papaya seed meal also contains a substantial amount of minerals that may, therefore, essential for the metabolism, body functions, and health of poultry [15]. Maisarah *et al*. [13] reported that papaya seed contains Ca 681, Mg 424, P 2,116, Fe 5.80, and Na 23.4 mg/100 g. Likewise, Moses and Olanrewaju [15] documented that papaya seed contains Ca 6.43, K 721, Fe 4.20, and Zn 6.41 mg/100 g. Previously, Adesuyi and Ipinmoroti [2] demonstrated that papaya seed meal contains Na, K, and Ca ranging from 33.6-16.2, 47.7-17.0, and 2.52-4.14 mg/100 g, respectively. Papaya seed also contains Mg, Zn, and Fe ranging from 0.53-2.81, 1.26-2.88, and 0.39-1.47 mg/100 g, respectively. The minerals Mn, Cu, Pb, and P were also found in papaya seed meal ranging from 1.11-1.27, 0.05-0.19, 0.00010-0.00013, and 28.5-58.6 mg/100 g, respectively. It is clearly shown from the above studies that there are variations in the minerals contents across the papaya seed meals. The different varieties or cultivars of the papaya seem to account for such divergent minerals contents in papaya seed, in addition to the ripening stages, soil conditions (where the papaya plants are cultivated), and climate conditions [2,10,17]. Apart from the potential of papaya seed as the mineral source, papaya seed meal also contains some anti-nutrient factors that may inhibit the absorption and utilization of nutrients including minerals by poultry. These anti-nutrient factors include oxalate, tannins, and phytate [2]. El-Safy *et al*. [12] documented that papaya seed meal contained phytic acid 23.3, tannins 10.6, and oxalate 1.89 mg/100 g dry weight meal. The papaya seed meal also contains trypsin inhibitors 1.77 TIU/mg protein. In general, the quantities of the anti-nutrient factors are relatively low, but the quantities may vary among the cultivars of papaya seed. For safety reason, the determination of anti-nutritional factors is, therefore, of importance before using papaya seed meal as poultry feed ingredient.

In general, plant-derived products are rich in vitamins, including papaya seed. In the study of Maisarah *et al*. [13], it was shown that papaya seed contains ascorbic acid 14.4 mg/100 g, β-carotene 120 μg/100 g, and Vitamin E 4.09 mg/100 g. Further, Chukwuka *et al*. [17] revealed that seed from ripe papaya contained Vitamin A 135 IU/mg, Vitamin C 14.7 IU/mg, riboflavin 0.02 mg, thiamine 0.03 mg, and niacin 0.11 mg. However, the contents of vitamins vary with the ripening stages of the papaya fruits. In the unripe condition, papaya seed contained Vitamin A 87.2 IU/mg, Vitamin C 11.7 IU/mg, riboflavin 0.01 mg, thiamine 0.05 mg, and niacin 0.10 mg [17]. On this basis, the use of seed from the ripe papaya seems to be more beneficial for the poultry, due to its higher vitamins contents.

## Papaya Seed as Protein Source for Poultry

Protein is one of the most important constituents in poultry rations as it serves vital metabolic functions, building and constructing of body tissues, repairing of body cells, and maintaining of good health [18]. Beyond its roles, protein is one of the most costly feed ingredients in poultry rations. Therefore, reducing the protein cost may be attributable to the reduced production cost of poultry farming. As presented in the earlier section, papaya seed contains a substantial amount of protein and may thereby be used as an alternative protein feed ingredient in poultry rations. With crude protein of 24-30% [2,3,10-16], papaya seed meal may be exploited as the alternative protein feed ingredient for poultry. In the context of protein digestibility, El-Safy *et al*. [12] reported that *in vitro* protein digestibility of papaya seed meal was 80.7%. It seems that the presence of anti-nutrient factors, particularly trypsin inhibitors and tannins, did not substantially inhibit the digestion and absorption of protein, as their quantities are relatively low in papaya seed [2].

Amino acids are essential components that are used to build body tissues of poultry [18]. Besides the high content of crude protein, papaya seed has also been reported to contain substantial amounts of amino acids (Table-2 [19,20]). With regard to lysine and methionine, these limiting amino acids (for poultry rations) seem, however, to be slightly lower in papaya seed meal as compared to those in canola meal and soy meal (Table-2 [19,20]). In general, the ripening stages may determine the content of amino acids in papaya seed meal. Oyeleke *et al*. [21] formerly noticed that amino acids, particularly lysine and methionine, were higher in the papaya seed from the ripe than those in the mature unripe papaya. However, the levels of lysine and methionine decreased in the seed of overripe papaya when compared with those in ripe papaya seed [21]. The latter authors further suggested that microbial activities taking place during the process of ripening seem to be responsible for the lower amino acids in the seed from the overripe papaya. The proportion of essential amino acids in feed ingredients is generally believed to determine the quality of protein feedstuffs. In general, protein feedstuffs should contain minimal 33% or more essential amino acids [22]. Indeed, the seed from mature unripe and ripe papaya contains about 48 and 47% of essential amino acids, respectively. In this regard, papaya seed could be regarded as a high-quality protein feedstuff for poultry. It should, however, be noted that papaya seed meal may only be included in broiler ration (to reduce the use of soybean meal) at a maximum of 5%, as the higher inclusion levels resulted in compromised growth performance and physiological conditions of broiler chickens [11]. In the case of the latter study, the fiber content seemed not to limit the inclusion level of papaya seed meal as the seed meal used contained only 1.67% of crude fiber. Bolu *et al*. [11] suggested that the presence of anti-nutritional factors in papaya seed may compromise the digestibility and thus feed utilization by the chickens. Actually, there are a number of studies exploiting papaya seed meal in poultry nutrition, but most of the studies used papaya seed meal as additive or supplement in feed [3,23,24], instead of using papaya seed meal as the substitute for protein-rich feed ingredients such as soybean meal for poultry.

**Table-2:** Amino acid profiles of papaya seed, canola meal, and soy meal.

Amino acids (g/100 g protein)	Papaya seed meal [19]	Canola meal [20]	Soy meal [20]
Lysine	4.21	5.80	6.40
Histidine	2.21	2.70	2.60
Arginine	6.44	5.80	7.20
Phenylalanine	3.38	3.80	5.00
Methionine	1.30	1.90	1.30
Threonine	2.85	4.50	4.00
Leucine	7.78	7.00	7.80
Isoleucine	3.09	4.00	4.00
Valine	2.25	5.00	4.80
Aspartic acid	7.05	7.00	11.7
Glutamic acid	12.4	17.5	18.7
Serine	3.01	4.60	5.10
Proline	2.13	6.00	5.10
Glycine	4.26	4.90	4.20
Alanine	3.22	4.30	4.30
Cystine	1.14	NM	NM
Cysteine	NM	1.70	1.60
Tyrosine	2.06	3.10	3.20
Tryptophan	NM	1.30	1.30

NM: Not measured

## Growth Promoting Effect of Papaya Seed on Poultry

In the post-antibiotic era, the presence of natural growth promoters for poultry is highly essential. Indeed, the retraction of antibiotics growth promoters from diets has been attributed to the retarded growth rate and thus poor economic performance of poultry farming. Although the studies are still limited, it was shown that papaya seed meal was able to improve the production performances of poultry, including increasing growth rate, egg production, and feed efficiency of poultry. In broiler chickens, Muazu and Aliyu-Paiko [3] showed that incorporation of 1% papaya seed powder in rations increased the final body weight and feed intake. In line with this, Rachmatika and Prijono [23] reported that incorporation of 1.2% papaya seed in diets increased body weight gain, reduced feed intake, and improved feed efficiency of *Raja* ducks. Likewise, Nideou *et al*. [25] documented that inclusion of 0.5, 1.0, and 2% papaya seed in the rations increased daily weight gain of pullet when compared with the control. In accordance with the growth-promoting effect, the administration of 0.5% papaya seed meal in diets increased egg weight, egg production, and feed conversion ratio of broiler breeder and improved day-old weight of chicks [24]. Moreover, Nghonjuyi *et al*. [26] reported that the administration of papaya seed extract at the levels of 480, 960, and 3200 mg/kg body weight increased body weight of *Kabir* chicks.

The definite mechanism by which papaya seed improved the growth performance of poultry remains unclear, but Muazu and Aliyu-Paiko [3] suggested that the improvement in organoleptic characteristics of the feed due to papaya seed administration seemed to increase feed intake and thereby growth performance of poultry. Furthermore, the improvement of gastrointestinal conditions and thus increased the digestibility and utilization of nutrients seemed to improve the production performance in poultry [25]. Further, the antimicrobial, anthelmintic, and antiparasitic activities [7,9,27] of the papaya seed were most likely to improve the gastrointestinal ecology and thus improve the health conditions and growth performance of poultry. Moreover, the contents of antioxidants [7] and minerals [15] in papaya seed seemed also to be responsible for alleviating the negative impact of stress and also improving the health of poultry. The latter conditions may, therefore, improve the growth performance of poultry. A recent study by Nghonjuyi *et al*. [26] further suggested that the presence of papain, chymopapain, caricain, and glycyl endopeptidase may improve the digestive process and thus enhance the growth rate of chickens. In the molecular level, papaya seed extract has also been reported to increase cell proliferation and reduce the apoptotic cells [28]. The latter condition seems, therefore, to enhance the growth rate of poultry.

## Antimicrobial and Antiparasitic Activities of Papaya Seed on Poultry

It is generally known that seeds of tropical fruits are rich in phytochemicals that may be essential to control and modulate the population of pathogens in the gastrointestinal tract of humans and animals. With regard particularly to papaya, the seeds from such fruit have been reported to contain alkaloids, flavonoids, steroids, saponins, papain, and terpenoids possessing antimicrobial as well as antiparasitic activities [4-6]. Indeed, several studies have reported the antimicrobial activities of papaya seed, for instance, Muhamad *et al*. [29] reported the antibacterial properties of papaya seed extract against *Salmonella enteritidis*, *Vibrio vulnificus*, *Proteus mirabilis*, and *Bacillus cereus*. Likewise, Peter *et al*. [30] showed the antibacterial activities of papaya seed toward *Staphylococcus aureus*, *Pseudomonas aeruginosa*, *Escherichia coli*, and *Salmonella typhi*. Similar to the previous study, Hidayati *et al*. [5] demonstrated the antibacterial activity of papaya seed against *E. coli* and *S. typhi*, while Masfufatun *et al*. [6] showed the efficacy of papaya seed against *Vibrio cholerae* and opportunistic pathogenic yeast *Candida albicans*. In accordance with this, the earlier study also showed the antifungal activity of papaya seed toward *Aspergillus flavus*, *C. albicans*, and *Penicillium citrinum* as reported by Singh and Ali [31].

Although a number of *in vitro* studies assessing the antimicrobial activities of papaya seed have been published, the *in vivo* study confirming such antimicrobial properties in poultry is, however, still lacking. The majority of *in vivo* studies more focus on the antiparasitic activities of papaya seed (in the forms of powder or extract), as presented in Table-3 [9,25-27,32-34]. It was apparent that papaya seed could serve as antiparasitic agents and thus may replace the use of synthetic antiparasitic drugs in poultry production. Ameen *et al*. [9] suggested that the presence of papain in papaya seed seemed to be responsible for reducing the parasites in the gastrointestinal tract of poultry as papain was able to digest bacteria and parasitic cells. Moreover, Kumar and Devi [4] revealed that the anthelmintic or antiparasitic properties of papaya seed are attributed to the presence of carpaine and carpasemine that have a wide spectrum antibacterial and antiparasitic activities. In addition, Nideou *et al*. [25] pointed out that benzyl isothiocyanate in papaya seed may inhibit energy metabolism and motility of parasites, while papain destroys the cuticle of parasites. In accordance, Zhang and Chen [35] reported that the benzyl isothiocyanate in papaya seed may also be responsible for the mitochondrial dysfunction of parasites. These conditions may result in the reduction of parasites in the gut of chickens. Furthermore, Jiménez-Coello *et al*. [36] revealed that fatty acid in the papaya seed may also decrease the number of parasites from both parasite stages, blood trypomastigote, and amastigote (intracellular stage). The daily administration of papaya seed may also avoid the ingestion of eggs by chickens, which can, therefore, prevent the eggs from growing and reach a mature stage in the gastrointestinal tract of chickens. In general, the antiparasitic effects of papaya seed on poultry are dose-dependent, in which higher level of papaya seed results in more reduced egg worms in the excreta [25]. However, it should be noted that high levels of papaya seed may negatively affect nutrient retention and thus production performance of poultry [11].

**Table-3:** Examples of studies using papaya seed as antiparasitic agents.

References	Results of studies
Ameen *et al*. [9]	Administration of papaya seed extract both in powdery (300 mg/day/bird) and aqueous (1:10 ml water required/day) reduced the fecal egg counts of *Heterakis gallinarum, Ascaridia galli,* and *Trichostrongylus tenuis* in commercial layers
Nideou *et al*. [25]	Daily feeding of papaya seed (1-2% of diets) reduced the population of helminths in the gastrointestinal tract of pullets
Nghonjuyi *et al.* [26]	Papaya seed extract (at the doses of 480,960, and 3200 mg/kg body weight) reduced fecal egg output of *A. galli* in *Kabir* chicks in Cameron
Dakpogan *et al*. [27]	Administration of papaya seed extract (1 mg per chicken for 5 days) was able to control the populations of *A. galli* and *Eimeria* sp. in free-range local breed chickens
Dougnon *et al*. [32]	Administration of papaya seed solution (2-4 g/L) reduced *A. galli* worms in layer Harco
Ozaraga *et al.* [33]	Papaya seed powder (6 g/kg body weight of chickens) reduced the egg per gram counts of roundworms of *Darag* native chickens
Feroza *et al*. [34]	Papaya seed extract (20 ml/kg feed) was capable of controlling *A. galli* infection in broiler chickens

## Antioxidative Activity of Papaya Seed on Poultry

In general, plants are known as good natural antioxidant sources that may be useful for poultry. Papaya seed has long been explored for its potential as natural source of antioxidants. The study by Kadiri *et al*. [37] clearly showed that papaya seed contains substantial amount of phenolic compounds such as ferulic acid, caffeic acid, p-coumaric acid, kaempferol-3-glucoside, p-hydroxybenzoic acid, and quercetin-3-galactoside. These make papaya seed be of nutraceutical importance in the poultry industry. In accordance with the above study, Salla *et al*. [38] reported that papaya seed is rich in polyphenols, flavonoids, alkaloids, tannins, and saponins. Moreover, Kumar and Devi [4] revealed that papaya seed contains lycopene, which highly reactive against free radicals. In addition to these, papaya seed also contains substantial amount of Vitamin C, Vitamin E, and β-carotene, which are good antioxidants [3,13]. The antioxidative properties of papaya seed have also been tested *in vitro*, in which Salla *et al*. [38] found that papaya seed could protect the HepG2 cells from oxidative stress. In line with this, Panzarini *et al*. [39] documented that papaya seed extract served as a potent free radical scavenger and protected the Detroit 550 fibroblasts undergoing H_2_O_2_ oxidative stress. In accordance, an earlier study by Nakamura *et al*. [40] documented that papaya seed extracted with n-hexane inhibited the generation of free radicals and apoptosis in HL-60 cells.

Recently, there is a growing interest among the poultry nutritionists to find the natural antioxidants as the alternative to chemical-based antioxidants. The use of chemical-based antioxidants is actually worried to induce a carcinogenic effect on humans as the consumers of poultry products [41]. In the very recent study, Muazu and Aliyu-Paiko [3] reported that feeding papaya seed powder (at the levels of 0.5 and 1.0% of diets) resulted in increased serum antioxidant activity (superoxide dismutase [SOD] and catalase [CAT]) and decreased lipid peroxidation in broiler chickens. In accordance with this, the study in mice by Sandhiutami *et al*. [42] showed that papaya seed extract increased the plasma level of SOD and decreased malondialdehyde (MDA) concentration, indicating the improvement of the antioxidative status of mice. Similar to this, Venkateshwarlu *et al*. [43] noticed the increased SOD, CAT, and glutathione and decreased MDA levels in the serum of rats with the administration of 200 and 400 mg/kg of papaya seed extract. It is very much likely that the above-mentioned antioxidative components in papaya seed are responsible for scavenging the free radicals (reactive oxygen molecules) and thus protecting the poultry from antioxidative stress. In addition, the presence of isothiocyanate in the papaya seed also seemed to inhibit the generation of superoxide (free radicals) in poultry [40].

## Immunomodulatory Activity of Papaya Seed on Poultry

The immune defense toward infections is crucial for maintaining the health of poultry. In the traditional human medicine, papaya seed has traditionally been used to improve the immune system of people [44]. In the *in vitro* study, Mojica-Henshaw *et al*. [45] showed that papaya seed extract increased the responsiveness of lymphocytes to phytohemagglutinin and inhibited the classical complement-mediated hemolytic pathway. In line with this, Amazu *et al*. [46] reported a substantial inhibition (57.1-64.2%) of papaya seed extract on the inflammation in rat model when induced with egg albumin. In accordance, Pathak *et al*. [47] revealed that papaya seed extract at the concentration of 10 mg/ml was able to inhibit the expression of interferon-gamma, tumor necrosis factor-alpha, interleukin-6, and nuclear factor-κB in pancreatic (HPDE-6) epithelial cells stimulated with methyl isocyanate. In commercial layers, Ameen *et al*. [9] reported that papaya seed extract improved the hematological and immune profile, that is, increased packed cell volume, red blood cells, hemoglobin concentration, and lymphocyte counts. The study in rabbits also documented that papaya seed meal increased the count of platelets, which are crucial components of the immune system [48]. To date, the study exploring the effect of papaya seed on the immune system of poultry is still lacking.

Several bioactive components have been suggested to play a pivotal role in immunomodulatory and anti-inflammatory activities of papaya seed. Kadiri *et al*. [7] suggested that enzymes, vitamins, polyphenols, and other active components are attributed to the pharmacological and immune-modulatory properties of papaya seed. In line with this, Pandey *et al*. [8] reported that enzymes (papain and chymopapain) and flavonoid in papaya seed could modulate the inflammatory markers that, in turn, regulate the immune responses and thus defense of host against pathogens. Likewise, Moses and Olanrewaju [15] revealed that papaya seed contains minerals, particularly zinc, that may be essential for the immune modulation of host. Furthermore, bioflavonoids in papaya seed may strengthen the host immune system [45]. It seemed that some bioactive compounds in papaya seed work in a synergistic manner to exert the immunomodulatory activities in host. This inference was actually supported by Mojica-Henshaw *et al*. [45] pointing out that there were no single components which are responsible for the immunomodulatory and anti-inflammatory activities of papaya seed.

## Conclusion

Papaya seed is agro-industrial by-product that is rich in protein and exhibits the growth-promoting effect, antimicrobial and antiparasitic activities, and immunomodulatory and antioxidative activities that are worthwhile as a functional feedstuff for substituting the role of antibiotic growth promoters for poultry. The in-depth study is needed to further elucidate the functionalities of papaya seed on poultry.

## Author’s Contributions

SS prepared and revised the manuscript. SS read and approved the final manuscript.

## Competing Interests

The author declares that he has no competing interests.

## Publisher’s Note

Veterinary World remains neutral with regard to jurisdictional claims in published institutional affiliation.
